# Fine-Scale Variability in Harbor Seal Foraging Behavior

**DOI:** 10.1371/journal.pone.0092838

**Published:** 2014-04-09

**Authors:** Kenady Wilson, Monique Lance, Steven Jeffries, Alejandro Acevedo-Gutiérrez

**Affiliations:** 1 Western Washington University, Department of Biology, Bellingham, Washington, United States of America; 2 Washington Department of Fish and Wildlife, Wildlife Science Program, Lakewood, Washington, United States of America; Texas A&M University-Corpus Christi, United States of America

## Abstract

Understanding the variability of foraging behavior within a population of predators is important for determining their role in the ecosystem and how they may respond to future ecosystem changes. However, such variability has seldom been studied in harbor seals on a fine spatial scale (<30 km). We used a combination of standard and Bayesian generalized linear mixed models to explore how environmental variables influenced the dive behavior of harbor seals. Time-depth recorders were deployed on harbor seals from two haul-out sites in the Salish Sea in 2007 (n = 18) and 2008 (n = 11). Three behavioral bout types were classified from six dive types within each bout; however, one of these bout types was related to haul-out activity and was excluded from analyses. Deep foraging bouts (Type I) were the predominant type used throughout the study; however, variation in the use of bout types was observed relative to haul-out site, season, sex, and light (day/night). The proportional use of Type I and Type II (shallow foraging/traveling) bouts differed dramatically between haul-out sites, seasons, sexes, and whether it was day or night; individual variability between seals also contributed to the observed differences. We hypothesize that this variation in dive behavior was related to habitat or prey specialization by seals from different haul-out sites, or individual variability between seals in the study area. The results highlight the potential influence of habitat and specialization on the foraging behavior of harbor seals, and may help explain the variability in diet that is observed between different haul-out site groups in this population.

## Introduction

Harbor seals are abundant marine predators throughout the northern hemisphere, yet we still have a limited understanding of their fine-scale behavior and ecological impacts in many regions. Although harbor seals dive for reasons unrelated to foraging (mating or resting at the bottom) diving is typically used as a proxy for understanding the foraging behavior of these mammals [1]–[3]. Modeling the fine-scale changes in dive behavior enables the prediction of future behaviors under varying environmental conditions. Further, understanding the variability of foraging behaviors within a population is important to fully understand the role of predators in the environment and identify the size and scale of specialization that may occur within a population or group of animals.

Harbor seals are ideal candidates for analyzing behavior on a fine spatial scale because the species has already been studied extensively around the world. Previous studies have revealed differences in diving behavior among different regional populations [4], and among age and sex classes [5]–[7]. They have also shown that harbor seals are opportunistic predators that feed on locally abundant prey and commonly switch foraging behaviors as prey abundances change seasonally and annually [8], [9]. Studies have also revealed species-wide similarities in optimal foraging depths [10], [11] and the importance of available habitat in determining foraging behavior [10], [12]. Most research has identified one foraging pattern throughout a respective study area. For example, Bjørge et al. [13] and Tollit et al. [12] found that harbor seals fed mostly on benthic prey with little diurnal variation in dive depths or types. In contrast, studies conducted in Alaska and Canada indicated that foraging occurred most often at dusk and that seals demonstrated a distinct diurnal foraging pattern, using square-shaped dives as they followed the diurnal vertical migration of prey [4], [14], [15]. These seasonal, spatial, and diel variations in behavior [7], [15], [16] show that foraging behavior differs both among and between populations and geographic regions (e.g. Sable Island, a single large haul-out site, and SE Alaska, multiple haul-out sites in the same region), but these findings do not address small-scale behavioral variation that may occur between haul-out sites within a population.

**Figure 1:**
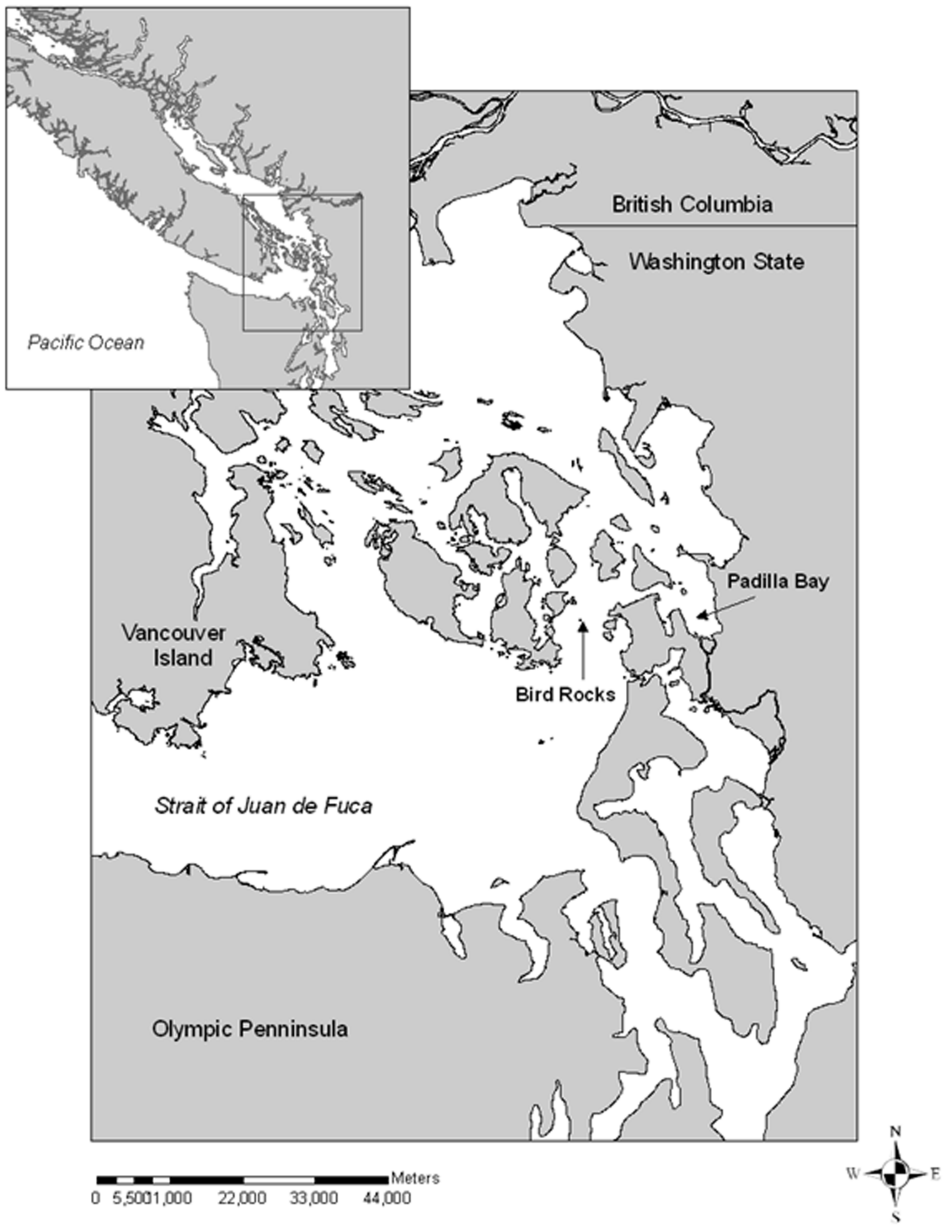
Harbor seal capture sites in the Georgia Basin. Inset: The study area in the Pacific Northwest: the Salish Sea. The Salish Sea encompasses Puget Sound, the San Juan Islands, the Canadian Gulf Islands, the Strait of Juan de Fuca and the Strait of Georgia.

The inland waters of Washington and British Columbia, a region known as the Salish Sea (Figure 1), are composed of diverse oceanographic and biological features. The Salish Sea includes three major basins: the Strait of Georgia, Puget Sound, and the Strait of Juan de Fuca, each with its own response to forcing mechanisms within the oceanographic system [17]. There are convoluted networks of islands, shallow tidal passes, estuaries, and a relatively large tidal range all within a restricted geographic area. The San Juan Islands are located near the convergence of all 3 of the major basins listed above, creating a dynamic and variable environment. Harbor seals are the only year-round resident pinnipeds in the Salish Sea and have an estimated population of 12 000 in the inland waters of Washington [18], and 39 000 in the Strait of Georgia in British Columbia [19]. There are numerous harbor seal haul-out sites throughout the San Juan Islands, where diet and population monitoring has been conducted since the late 1970s [18], [20], [21]. These sites tend to fall into two broad categories: estuarine (soft-bottomed bays) and non-estuarine (rocky-reef islands) [22], [23]. The presence and abundance of prey species in certain areas is typically correlated with the habitat available and the sediment type [24]. Therefore, the variation in prey associated with different haul-out sites (rocky vs. sandy) in the region, suggests that the foraging behavior and diet of harbor seals may vary between site types. Given the diverse and dynamic ecosystem and the ubiquity of harbor seals in the area, the San Juan Islands is an excellent system in which to study the variability of foraging behaviors within a population of predators.

Harbor seals in the San Juan Islands can move long distances (>100 km) and haul-out in multiple locations; however, they tend to travel to and from a single site and appear to be faithful to these locations [25]–[27]. Identifying differences between haul-out site groups, may be indicative of individual variability; however, it may also indicate prey or habitat specialization within the study area [28]. In the second case, seals may have adopted distinct foraging strategies depending on where they haul-out and which prey is readily available, even though seals hauling out less than 5 km away, in an area they could easily exploit, have adopted different strategies for use in a different habitat. The primary goal of this study was to identify differences in diving behavior between relatively close haul-out sites (∼20 km apart). We hypothesized that foraging behavior in the San Juan Islands was site-dependent due to the dynamic and variable ecosystem and the diversity of available prey between haul-out sites. We tagged seals from two different haul-out sites in the San Juan Islands to examine this hypothesis and to describe the variability of diving behavior within this population and identify differences in foraging behavior for harbor seals on a small spatial scale.

## Materials and Methods

### Ethics Statement

This study was conducted in accordance with animal use protocols reviewed and approved by the Institutional Animal Care and Use Committee at Western Washington University (Protocol Number 06-005) and at the National Marine Mammal Laboratory. All research and animal handling was conducted under the Marine Mammal Protection Act Scientific Research Permit 782–1702 awarded to the National Marine Mammal Laboratory by the NOAA Office of Protected Resources for scientific research.

### Study Site

Harbor seals were captured in the spring of 2007 and winter of 2007–2008 at two sites in the San Juan Islands: Padilla Bay National Estuarine Research Reserve (hereafter Padilla Bay) (n = 15) and Bird/Belle Rocks (n = 14) (Figure 1). Padilla Bay is a large soft-bottomed estuarine bay situated near the mouth of the Skagit River (center at 48°28.37′N, 122°30.88′W). Bird and Belle Rocks are rocky reef, non-estuarine haul-out sites located in Rosario Strait. Bird Rocks (48°29.16′N, 122°45.16′W) is a congregation of three rocky reef islands used year-round by harbor seals and during the winter months by Steller sea lions (*Eumetopias jubatus*). Belle Rock is a rocky reef located <1 km from Bird Rocks and is exposed only during low tide. Given their close proximity, data from Bird and Belle Rocks were combined for analysis and will from now on be referred to only as Bird Rocks. Both Bird and Belle Rocks are located roughly 20 km from Padilla Bay and seals are capable of moving between the two locations.

### Instrument Deployment

Adult harbor seals were captured from April–May of 2007 and from November 2007–February 2008 following the methods of Jeffries et al. [29]. After capture, weight to the nearest 0.5 kg and straight length ±5 cm was measured, blood and blubber biopsies were taken, and time-depth recorders (TDRs; Wildlife Computers, Redmond, WA, Mk-9, or Mk-10F) were attached to each seal. Tags were glued to the pelage of the animal, along the dorsal midline between the shoulders, using 5-min epoxy (ITW Devcon, Danvers MA).

Tags were programmed to record data every day for the duration of the study and were expected to come off with the annual molt. TDRs were set to record time, pressure (depth), light level, and temperature (tag temp. for Mk-9, water temp. for Mk-10F) every 10 sec [15], [30], and were equipped with an Eco-tech floatation pack with a VHF transmitter. The instrument package was positively buoyant and balanced to float with the VHF antenna upright to allow for tracking and recovery by boat after it became detached during the seals annual molt between August and October of either 2007 or 2008. Permit constraints required all instrument packages to weigh ≤1% of each tagged seal's body mass. This weight requirement is within the range recommended by Macdonald [31] and Brooks *et al* [32] to avoid adversely influencing the normal behaviors of instrumented animals, and is similar to that employed in other studies of harbor seal behavior [14], [28], [30], [33]. Additional details on capture and tagging can be found in Peterson [27].

### Data Processing

The study period was roughly 2 years in duration. Due to variable molt times, and the number of seals tagged at different times of the year, few seals retained their tags through the duration of the study. Therefore, data for each seal were visually inspected to designate a date where diving behavior was observed to end (i.e. if the tag fell off while the animal was on land, or the tag was floating in the water for days). Analysis of movement patterns of the tagged animals showed that although some rocky reef seals traveled great distances, all of the seals returned to their respective tagging sites throughout the study duration [25], [27]. During this study, seals from Bird Rocks traveled close to Padilla Bay, but were not observed (foraging or hauled-out) within the estuary. Both groups remained faithful to the locations where they were tagged. This behavior suggested that pooling seals by their tagging sites accurately identified where they were hauling-out during this study. Data were downloaded and processed using software provided by Wildlife Computers (Redmond, WA). All dives were corrected using Zero-offset correction software to account for drift in the TDRs pressure transducer, which estimates water depth. The resolution of the pressure transducer was 0.5 m with an accuracy of ±1% of the depth reading; however, previous studies examining dive classification techniques show that dives with at least five depth readings, regardless of sampling, provide the most precise representation of dive shapes [34]. In this study, the number of dives with less than five readings increased dramatically for dives ≤5 m and were therefore excluded from analysis because we were using dive shape for classification purposes following Lesage et al. [14] and Baechler et al. [30].

Wildlife Computers' dive analysis software (v.1.0.55) was used to analyze the corrected dive records and to classify the following variables for each dive: maximum depth, duration, bottom time (time spent ≥85% of the maximum depth of the dive), wiggles (the number of vertical movements within the bottom portion of the dive), and average ascent and descent rates. Four additional variables were used for dive classification: skew (the ratio of average ascent rate to average descent rate), the ratio of bottom time to dive duration (BTD), the ratio of bottom time to maximum depth (BTM), and the ratio of maximum depth to dive duration (MDD) [14], [30], [35].

### Dive Classification

Diving by air-breathing marine predators, such as harbor seals, can be viewed as excursions from the surface to search for and/or consume prey [36] and can be characterized by multiple factors. Dive shapes for harbor seals typically fall into one of two broad categories: square or V-shaped, and within these categories other factors such as skew, wiggles, and depth are used to identify more specific shapes and inferred behaviors. In this study, eight variables were used to classify dives and determine dive shapes: 1) maximum depth, 2) duration, 3) bottom time, 4) wiggles, 5) skew, 6) BTD, 7) BTM, and 8) MDD. Wiggle count was a defining characteristic of wiggle-dives and was therefore deemed categorical, where all dives with a wiggle count >0 were considered wiggle dives. To generate a smaller set of uncorrelated variables, the eight numerical dive variables were initially analyzed using a Principle Components Analysis (PCA). The principal components (PCs) that accounted for ≥80% of the variance were used in subsequent analyses. The resultant factor scores from the PCA, plus the wiggle-dive variable, were then introduced into a k-means cluster analysis [14], [37], [38]. Previous studies have identified between five and seven dive types for harbor seals [14], [30]. The optimum number of clusters for this study was determined by analyzing the cluster solutions for four to eight clusters. The cluster solutions were validated using a discriminant function analysis [14], [35], [37] and the appropriate number of clusters was determined as the most parsimonious solution; the one with the fewest number of clusters and the highest percent classification accuracy. Multiple studies have inferred behaviors for dive shape based on the combination of dive records with stomach temperature telemetry [14], [36], [39] or Crittercam video recorders [40]–[42]. Due to the increased proportion of time spent at depth, square-shaped dives are typically considered foraging dives, while V-shaped dives are associated with traveling or exploratory behavior [14], [34], [43]. A square dive with wiggles suggests vertical movements in the bottom portion of the dive and potential feeding within a prey patch, square dives without wiggles may suggest benthic foraging or searching for prey [35], [44], [45].

### Bout Classification

Harbor seals rarely forage using individual dives, but perform a series of consecutive dives while working a particular area [16]. To develop a more biologically relevant measure of behavior for this species we grouped individual dives into bouts of diving [2], [36]. We used a modified version of Boyd's [2] iterative statistical method to identify these bouts. Following Boness et al. [16], we operationally defined the beginning of a diving bout as a minimum of four consecutive dives to at least 6 m. After the start of a bout, subsequent dives were added if the next surface interval was not significantly greater than the mean surface intervals from the previous dives within the bout according to a t-test with an alpha value of 0.05 [1], [2]. The bout ended when the subsequent surface interval was significantly greater than the previous surface intervals within the bout.

Bouts were classified using eight variables: 1) number of dives within the bout, 2) mean dive depth, 3) mean dive duration, 4) mean surface interval, 5) bout duration, 6) percent of time spent at depth, 7) percent of square-shaped dives, and 8) percent of V-shaped dives. The same procedure described to classify individual dives was used to classify diving bout types. Bout variables were analyzed using a PCA to produce a smaller set of orthogonal variables and then PCA factor scores were assessed using a k-means cluster analysis. The cluster solutions were validated using a discriminant function analysis and the most parsimonious cluster solution was accepted. Bouts with a high proportion of square-shaped (foraging) dives were considered foraging bouts.

### Model Selection

We used a Bayesian approach to analyze dive behavior because it provides a means of synthesizing data from multiple sources and serves as a tool for updating our knowledge based on observed data [46]. Modeling dive behavior in this way allowed us to incorporate prior knowledge about behavior and then identify the influence of different predictors such as site, season, light, or sex on said behavior using the posterior. A Bayesian approach is potentially more applicable as a tool in ecology because inference is drawn from the posterior predictive distribution instead of the data themselves, which may be biased.

We analyzed variation in dive behavior by comparing seasonal, temporal, and sexual variation in the occurrence of different diving bouts between haul-out site groups. The different life history stages that occurred during the study period were divided into breeding (July–October) and non-breeding (November–June) seasons based on seal pupping and molting phenology in the San Juan Islands [47], [48]. The breeding season defined here includes both pupping and breeding behaviors, and the non-breeding season includes post-molt/winter behaviors. The molting season was excluded from the analysis because the seals would have lost their tags shortly after beginning to molt and during the winter deployment we only tagged seals that had already completed their molt. Therefore, we do not believe the data represent molting behavior for any of the tagged individuals. Light categories were determined using the mean sunrise and sunset times for each month during the study and assigning a dive as occurring during the day or at night. To determine which combination of predictors to include in the final model a Generalized Linear Mixed Model (GLMM) with a binomial sampling distribution was run on the data using the glmer() function in R [49]. The fixed factors included in the model were haul-out site, season, sex, and light, individual seals were included as a random effect. Initial variable selection was performed by comparing BIC and AIC values for a suite of models. The model with the lowest BIC and AIC values was chosen as the best fit. The factors that significantly influenced the subset of data used in model selection were then included in the final design matrix (X). We used a Gibbs sampler to model the data with haul-out site, season, sex, and whether it was light or dark outside (light) as predictor variables.

In the final model, we used a binomial likelihood with an uninformative normal prior distribution. A vague prior was used to allow the data to dominate the analysis and dictate the shape of the posterior distribution, while still accounting for the high levels of variability in harbor seal diving. The model setup was:
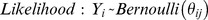
(1)

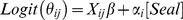
(2)Y_i_ is the response variable representing which bout types the seal performs (Type I or Type II). θ_ij_ is the probability associated with the binomial distribution for seal *i* and dive *j*, X is a design matrix of predictor variables, β refers to the coefficients associated with each predictor (the effect of each predictor in determining what behavior is being performed), and α is the coefficient associated the random effect (individual seals). A metropolis step was added within the Gibbs loop to accept or reject proposed β values during each iteration of the model. The priors on β and α were normal distributions with starting values specified as follows:
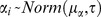
(3)

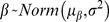
(4)All analyses were performed with R version 2.12.

## Results

Twenty nine seals were tagged in the San Juan Islands during two deployment periods. Eleven of the fifteen TDR packages deployed in Padilla Bay were recovered and ten of the fourteen packages from Bird Rocks were recovered. One seal from Padilla Bay was tagged during both deployment periods. Most adult females were believed to be pregnant and were expected to have given birth and nursed a pup following instrument deployment. Most seals retained their tags for at least 3 months, although some animals recorded data for 6–7 months. Data were collected from April 2007 through August of 2008. A total of 326,869 dives were recorded for 11 adult females and 10 adult males (Table 1).

**Table 1:** Data collected from each tagged harbor seal relative to capture site in the San Juan Islands.

Site and ID	Sex	Mass (kg)	TDR	# Dives	Retention (d)	Tagging Trip
Bird Rocks						
B1695	M	71.5	Mk-10	38,201	152	Spring
B1696	M	74.5	Mk-10	14,322	56	Spring
B1700	M	86.0	Mk-9	9,472	52	Spring
B1701	M	81.5	Mk-10	42,888	164	Spring
Y1455	F	76.5	Mk-10	13,277	87	Spring
B1742	M	83	Mk-10	5,101	33	Winter
B1744	M	81.5	Mk-10	13,798	93	Winter
B1745	M	83	Mk-10	8,623	51	Winter
Y1513	F	75.5	Mk-9	26,371	147	Winter
Y1514	F	70.5	Mk-9	34,660	182	Winter
**Total**	**7M, 3F**			**206,713**		
Padilla Bay						
B1699	M	64.0	Mk-9	5,441	95	Spring
B1712	M	69.0	Mk-9	2,218	71	Spring
B1713	M	54.0	Mk-9	3,203	64	Spring
Y1456	F	55.5	Mk-9	4,253	93	Spring
Y1457	F	57.5	Mk-9	17,136	97	Spring
Y1458	F	48.5	Mk-9	14,201	118	Spring
Y1459	F	83.0	Mk-9	44,583	112	Spring/Winter
Y1460	F	62.5	Mk-9	8,106	103	Spring
Y1462	F	77.5	Mk-9	1,164	69	Spring
Y1465	F	103.0	Mk-9	14,709	78	Spring
Y1469	F	85.0	Mk-9	5,142	137	Spring
**Total**	**3M, 8F**			**120,156**		

### Dive Classification

**Figure 2:**
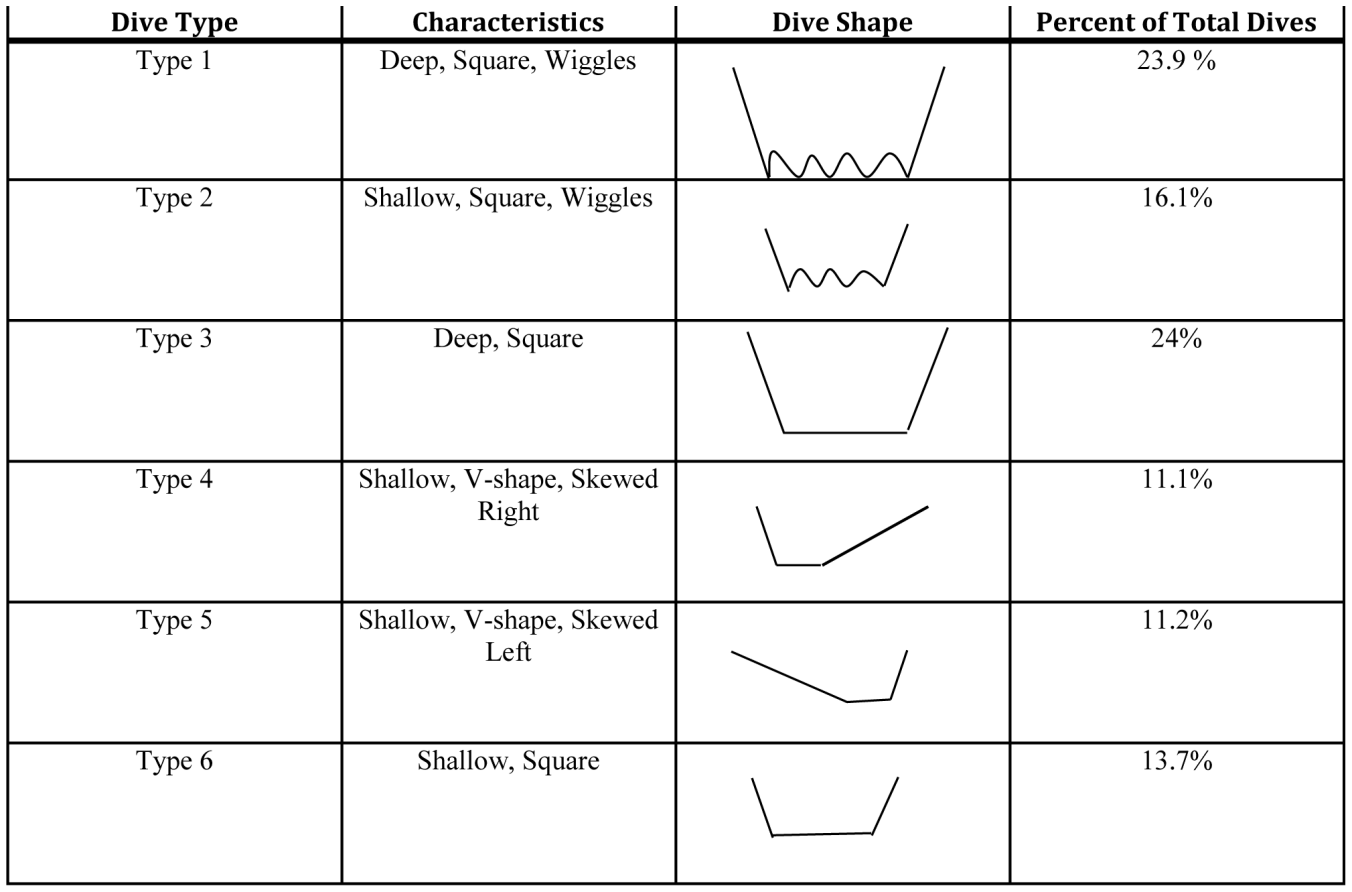
Dive shapes and general characteristics of harbor seal dives in the San Juan Islands. Deep dives are those with a maximum depth ≥20 m, shallow dives are <20 m.

A total of 297,964 dives were classified. All of the dive variables loaded significantly on at least one PC and were therefore included in subsequent analyses. K-means cluster analysis resulted in six dive types with 95.2% classification accuracy. The six clusters were assigned a dive type after visual inspection of the results (Figure 2). Four of the six dive types were square-shaped, with a mean bottom time ≥50% of the total dive duration. The remaining two dive types were considered V-shaped with a mean bottom time ≤37% of the total dive duration (Table 2). Type 1 and type 3 dives were considered deep dives (≥20 m) and were similar in depth, duration, and bottom time; however, type 1 dives were classified as wiggle dives and type 3 dives were not (Figure 2). Dive types 2 and 6 were considered shallow dives (<20 m) and were also separated by wiggles (Figure 2, Table 2). Dive types 4 and 5 (the V-shaped dives) were both considered shallow (<20 m) with similar durations and differed in skew. Type 4 dives were skewed to the right indicating a slow ascent rate compared to descent rate, and type 5 dives were skewed to the left indicating a slower descent rate. On average, type 4 and type 5 dives were shorter in duration than all the square-shaped dives.

**Table 2:** Mean (± SD) values of harbor seal dive types in the San Juan Islands.

	Type 1	Type 2	Type 3	Type 4	Type 5	Type 6
Max Depth (m)	62±30.65	13.36±7.19	56.23±23.51	17.55±12.8	16.13±12.68	13.57±7.71
Duration (s)	310.14±107.69	219.27±99.57	304.58±78.48	148.73±73.77	166.45±73.92	241.79±106.89
Bottom Time (s)	181.62±96.69	152.81±89.11	192.14±76.16	39.51±29.53	60.03±46.45	179.79±100.31
Wiggles	1.05±0.38	1.04±0.29	0±0	0.46±0.51	0.35±0.48	0±0
Skew 1	0.94±0.26	1.01±0.48	0.95±0.23	1.8±1.17	0.47±0.17	1.04±0.48
Skew 2	1.14±0.36	1.18±0.51	1.11±0.26	0.71±0.29	2.64±2.54	1.12±0.43
Avg Descent Rate (m s^−1^)	1.11±0.33	0.54±0.26	1.1±0.33	0.42±0.27	0.65±0.67	0.52±0.26
Avg Ascent Rate (m s^−1^)	1±0.3	0.5±0.25	1.02±0.33	0.61±0.37	0.29±0.18	0.5±0.25

### Diving Bouts

A total of 45,013 diving bouts were identified with an average duration of 35.17±0.17 min. Three bout types were classified with 97.60% classification accuracy. Bouts were broadly separated by mean dive depth and bout duration (Table 3). Type I bouts were classified as both long and deep (≥20 m) and were composed of mostly square-shaped dives (95%). Type II bouts were composed of both square and V-shaped dives, but were shallower in depth and shorter in duration than Type I bouts. Type III bouts made up ≤1% of the bouts used in this study. They were shallow in depth, long in duration, and contained mostly square-shaped dives; however, the bottom time for these bouts only accounted for 3.9% of the total bout duration. The average surface interval of type III bouts was extremely long (∼2 hr) indicating that these bouts potentially represent surface resting or other behaviors not associated with foraging. Due to the high proportion of square-shaped dives in both Type I and II bouts, both were considered potential foraging bouts, with differences in the depth at which foraging occurred and potentially the type of prey being consumed. Since we were interested in the variability of foraging behavior, and Type III bouts were likely non-foraging bouts and also rarely used, only Type I and Type II bouts were used in the final analyses.

**Table 3:** Mean (± SD) values of harbor seal bout types in the San Juan Islands.

	Type I	Type II	Type III
Number of Dives	5.82±2.71	5.63±2.35	5.47±3.11
Dive Depth (m)	44.73±27.45	18.97±11.47	18.86±12.84
Dive Duration (s)	288.93±84.88	165.59±57.57	193.34±81.54
Surface Int. Duration (s)	45.46±28.25	39.22±61.37	3139.46±2254.39
Bout Duration (s)	2267.70±1071.29	1355.34±739.92	20214.87±12765.20
Time at Depth (%)	0.55±0.13	0.33±0.13	0.04±0.03
Square-shaped Dives (%)	0.95±0.09	0.44±0.24	0.53±0.28
V-shaped Dives (%)	0.05±0.089	0.56±0.24	0.47±0.28

### Model Selection

The model was initially run with simulated data to verify its structure and the results. Due to the complexity of the model and our uncertainty on where to initialize the prior, the sampler was run hundreds of thousands of times on a subsample of data before we reached acceptable convergence. The final model was run using the entire dataset, initialized at mean values from the previous run, and then run for 1,000,000 iterations.

**Figure 3:**
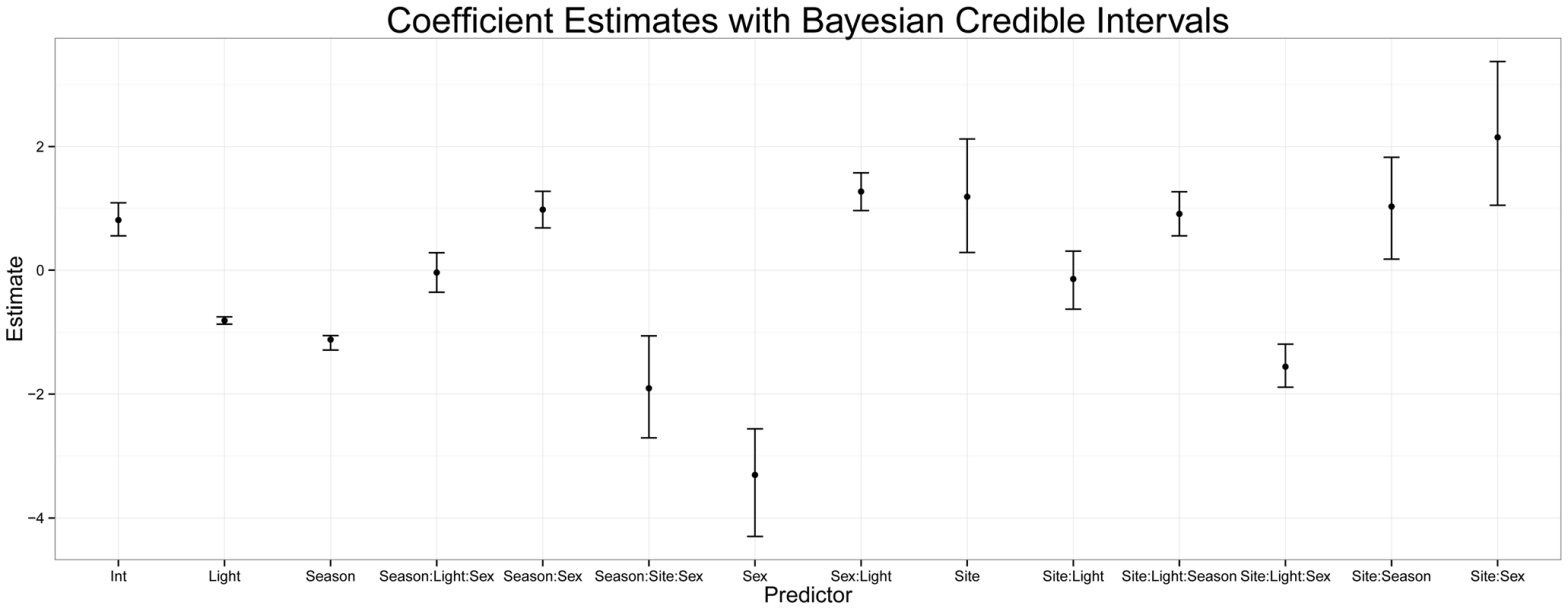
Beta coefficient estimates for predictor variables.

Variable selection revealed that the full interaction model had the best fit to the data with haul-out site, season, sex, and light (day/night) all influencing dive behavior (Table 4). Nearly all of the fixed and random effects had a positive or negative effect on behavior (Tables 5 & 6). A positive effect indicates a shift from Type I to Type II bouts with a positive unit change in the predictor; a negative effect is the opposite, with the shift being from Type II to Type I. Variables with a credible interval spanning across zero did not have a significant effect on dive behavior, but still helped explain some of the residual deviance in the model (Figure 3).

**Table 4:** Model output for GLMM after initial model selection.

Predictor	Estimate	Std. Error	Z value	Pr (>|z|)
Intercept	0.94	0.33	2.89	<0.01
Site 1	1.06	0.77	1.38	0.17
Season 1	−1.79	0.07	−25.66	<2e-16
Light 1	−1.40	0.06	−22.70	<2e-16
Sex 1	−3.27	0.61	−5.40	6.77e-8
Site1:Season1	1.96	0.53	3.71	<0.01
Site1:Light1	1.05	0.81	1.29	0.20
Season1:Light1	0.91	0.08	11.98	<2e-16
Site1:Sex1	1.99	0.97	2.05	0.04
Season1:Sex1	1.64	0.17	9.40	<2e-16
Light1:Sex1	1.86	0.18	10.55	<2e-16
Site1:Season1:Light1	−0.69	0.83	−0.83	0.41
Site1:Season1:Sex1	−2.83	0.56	−5.10	<0.01
Site1:Light1:Sex1	−2.76	0.83	−3.32	<0.01
Season1:Light1:Sex1	−0.96	0.19	−4.92	<0.01
Site1:Season1:Light1:Sex1	1.64	0.85	1.92	0.06

**Table 5:** Posterior output for the fixed effects in the model examining harbor seal dive behavior in the San Juan Islands.

		95% Credible Interval	
	Mean	0.025	0.975
Intercept	0.811	0.556	1.091
Site	1.188	0.287	2.124
Season	−1.118	−1.287	−1.053
Sex	−3.309	−4.298	−2.563
Light	−0.810	−0.868	−0.750
Site:Season	1.03	0.179	1.828
Site:Sex	2.15	1.051	3.371
Season:Sex	0.98	0.684	1.277
Season:Sex:Site	−1.906	−2.708	−1.057
Site:Light	−0.143	−0.625	0.308
Sex:Light	1.272	0.965	1.575
Site:Light:Sex	−1.557	−1.889	−1.192
Site:Light:Season	0.911	0.556	1.270
Season:Light:Sex	−0.039	−0.358	0.282

Values are shown on a logit scale for the influence of each predictor on seal behavior.

**Table 6:** Posterior output of the random effects in the model.

		95% Credible Interval	
Seal ID	Mean	0.025	0.975
B1695	−0.261	−0.634	0.013
B1696	0.269	−0.101	0.560
B1699	−0.647	−1.340	0.028
B1700	−0.435	−0.807	−0.129
B1701	0.189	−0.186	0.460
B1712	0.898	0.064	1.752
B1713	0.300	−0.456	1.052
B1742	−0.821	−1.217	−0.473
B1744	−0.415	−0.786	−0.114
B1745	−1.354	−1.740	−1.030
Y1455	−0.640	−1.281	0.338
Y1456	1.249	0.790	1.729
Y1457	−1.186	−1.597	−0.746
Y1458	−0.409	−0.823	0.034
Y1459	−1.240	−1.643	−0.806
Y1460	1.759	1.319	2.230
Y1462	−1.416	−2.049	−0.801
Y1465	−0.499	−0.909	−0.060
Y1469	0.842	0.386	1.319
Y1513	1.044	0.416	2.036
Y1514	0.154	−0.476	1.123

Values are shown on a logit scale for the effect of each individual seal.

**Figure 4:**
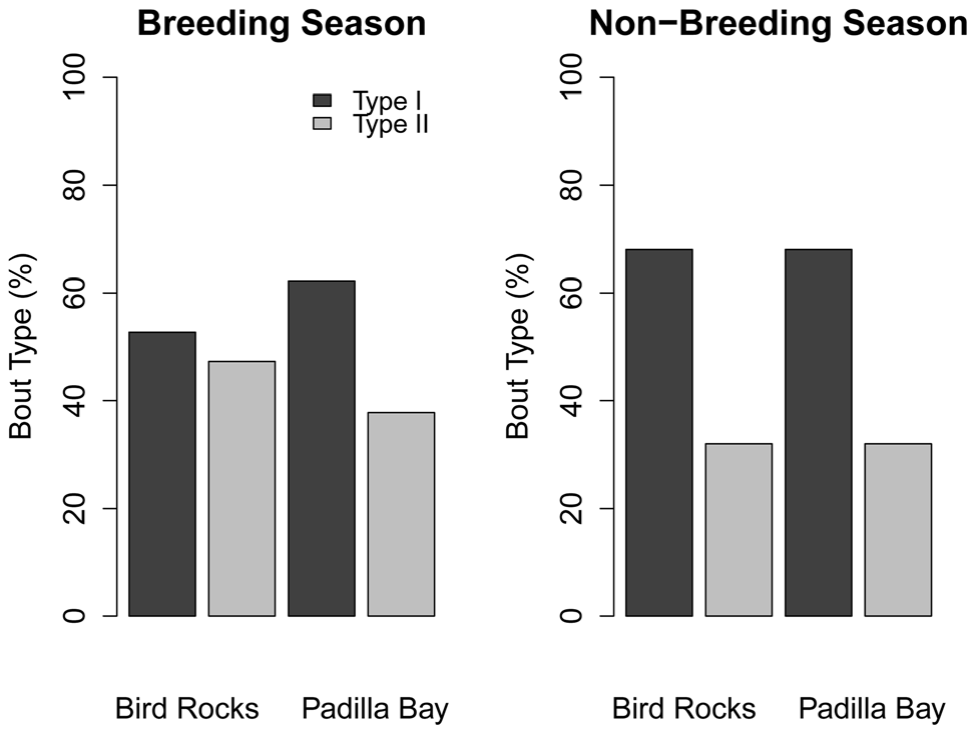
Distribution of harbor seal bout types relative to season and haul-out site. The breeding season is July–October and the non-breeding season is November–June.

**Figure 5:**
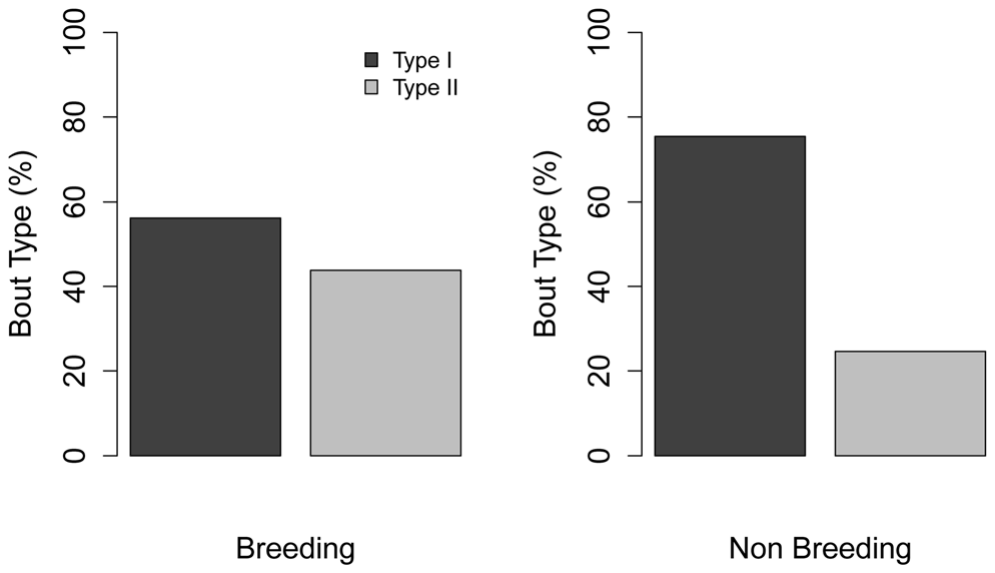
Distribution of harbor seal bout types relative to season.

Haul-out site had a significant influence on behavior (Table 5), which became evident when looking at how the interactions of other predictors with haul-out site influenced behavior. The interaction of haul-out site and season showed more Type I bouts occurring at both sites during the non-breeding season (Figure 4). During the breeding season, there was an increase in Type II bouts at both sites, but seals from Padilla Bay still performed a higher proportion of Type I bouts than seals from Bird Rocks (Figure 4). Differences were also observed when looking at season alone. Although more Type I bouts were used in both seasons, the proportions of Type I and Type II bouts were nearly equal during the breeding season, whereas Type I bouts were nearly 3× higher and Type II bouts decreased by half during the non-breeding season (approximately 80% vs. 20%) (Figure 5).

**Figure 6:**
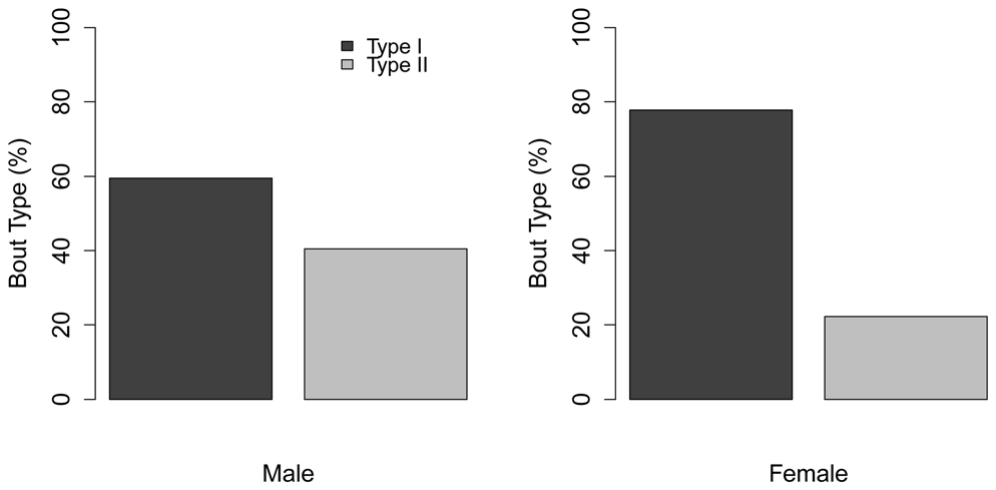
Distribution of harbor seal bout types relative to sex.

**Figure 7:**
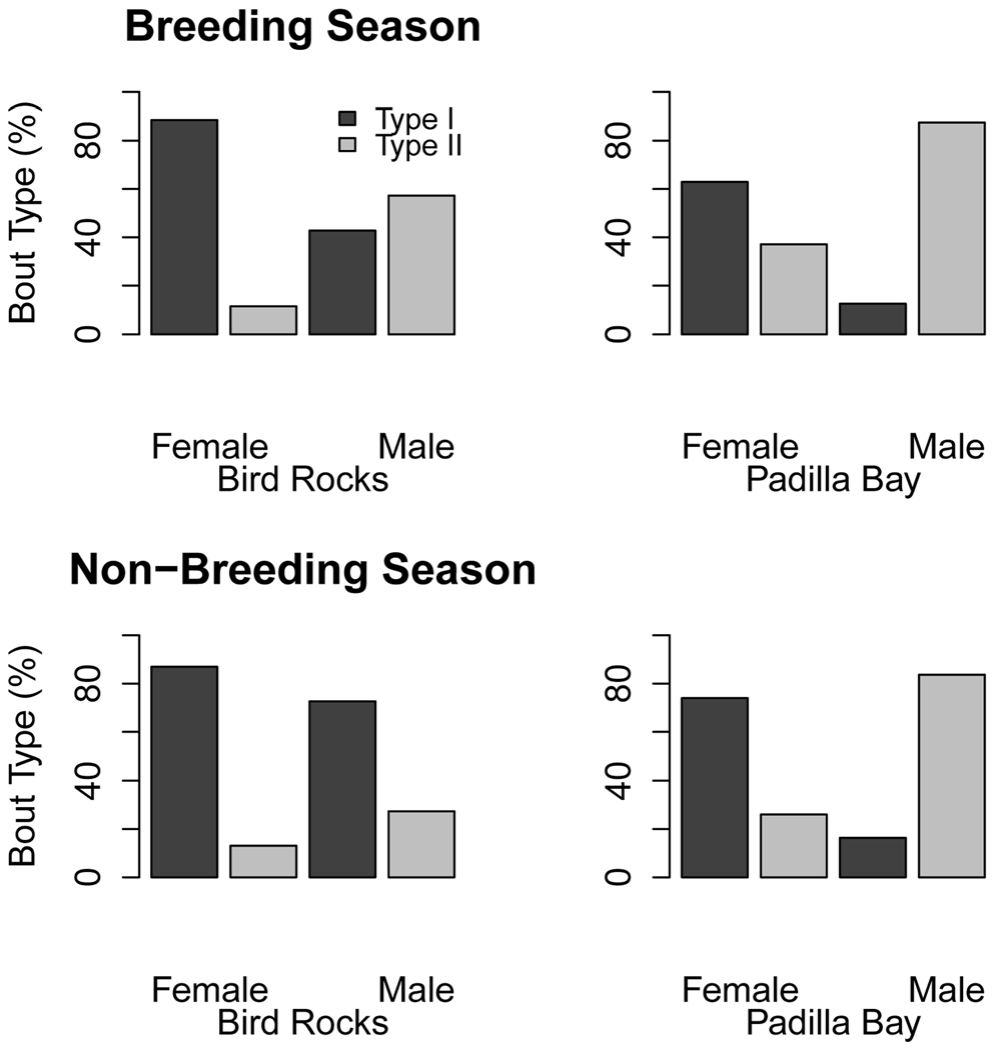
Distribution of harbor seal bout types relative to haul-out site, season, and sex. The breeding season is July–October and the non-breeding season is November-June.

The same pattern, with changes in the proportional use of bout types, was visible with the influence of sex on dive behavior (Figures 6 & 7). Type I bouts were the predominant type used by both sexes, but females used significantly more of the deep, Type I bouts than the shallower, Type II bouts, while males used similar proportions of both. However, males from Bird Rocks were the only ones to significantly shift their behavior. During the breeding season the proportions of bout types used were nearly equal, with slightly more shallow diving (Type II bouts); in the non-breeding season they switched their behavior to significantly more deep diving (80% of bouts were Type I, Figure 7).

**Figure 8:**
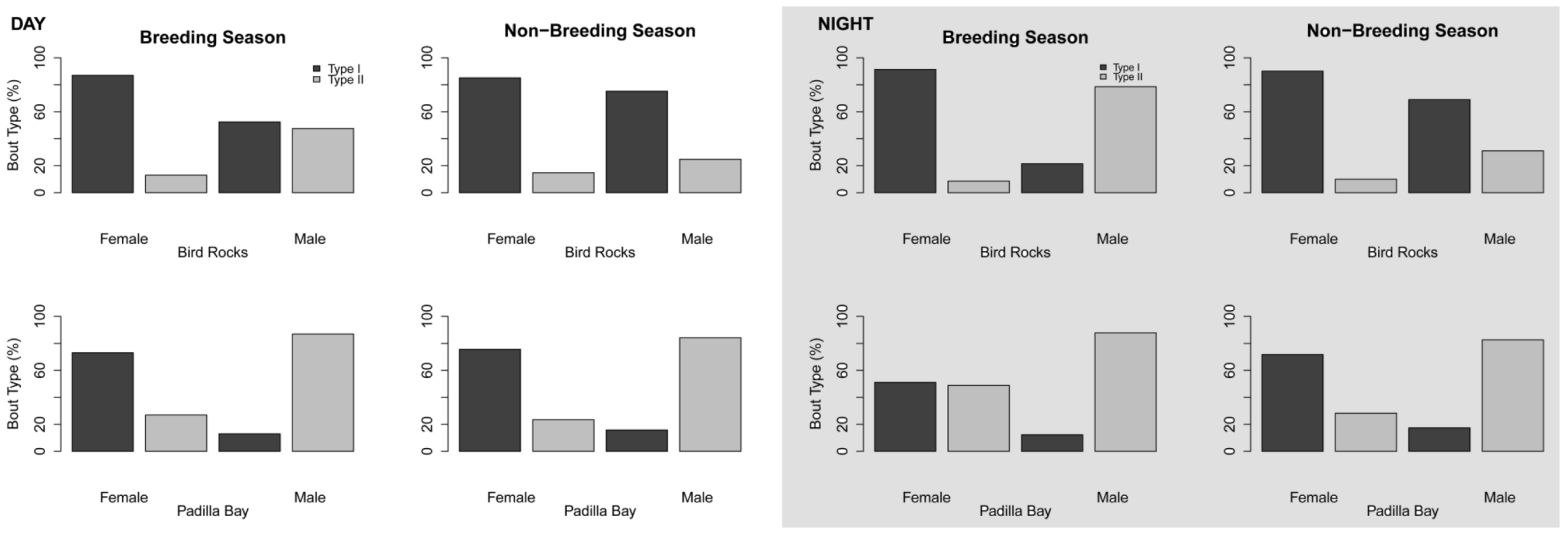
Distribution of harbor seal bout types relative to light, site, season, and sex. The breeding season is July–October and the non-breeding season is November–June.

The effect of light on dive behavior was most apparent when looking at the full interaction (haul-out site, season, and sex (Figure 8)). A greater percentage of Type II bouts were performed at night during the breeding season. During the day, at both sites during both seasons, Type I bouts were the predominant bout type used. During the breeding season, males at Bird Rocks increased their use of Type II bouts from equal proportions to more Type II bouts at night, and females at Padilla Bay increased their use of Type II bouts from more Type I to almost equal proportions at night. Light did not appear to affect the other sexes at each site. During the breeding season, there was little change in dive behavior for either sex or site between day and night.

**Figure 9:**
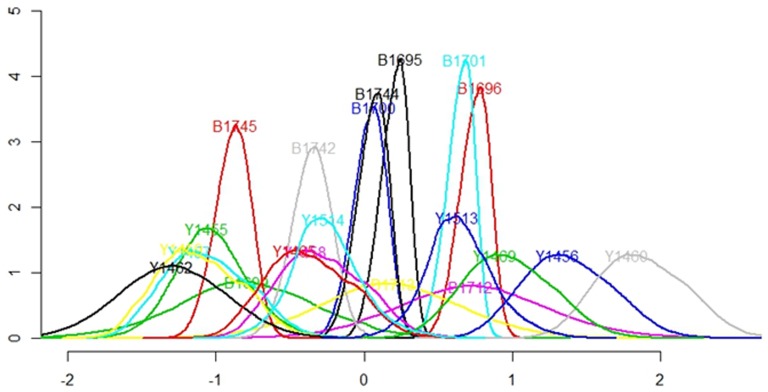
Density plot of the random effects in the model. A peak near −1 indicates that the individual performed more Type I bouts, a peak near +1 indicates more Type II bouts, and a peak near 0 indicates no distinct difference in the use of different bouts for that individual.

The results also revealed significant random effects for nearly every individual seal (Table 6). The spread of behavioral variability both among and within individuals is apparent in Figure 9. A positive peak demonstrates a tendency of that individual to perform Type II, shallow, bouts, while a negative peak shows a preference for deeper, Type I bouts. Individuals with a peak density near zero did not show a strong preference between the different behaviors during the study period.

## Discussion

All of the predictors used in this study significantly influenced the dive behavior of harbor seals in the San Juan Islands. This result suggests that where seals haul out, whether or not they are breeding, whether it is light or dark outside, or if they are male or female all affect their dive behavior in some way. Harbor seals are opportunistic predators that adjust their foraging behavior according to prey availability and profitability [8], [9]. The seasonal variation in bout use observed in this study suggests that seals may have switched the predominant prey in their diet during different seasons. Diet studies in the San Juan Islands indicate that harbor seal diet varies according to prey migrations and that different haul-out sites may focus on different prey species at different times of year [20], [28]. The variations observed by site and sex suggest that individuals may be exhibiting prey specialization or habitat exploitation. Indirect evidence (via diet studies) of prey specialization for harbor seals in the study region provides support for the first hypothesis [20], [50].

### Dive Classification

Studies examining pinniped dive behavior have usually identified five to seven dive types [2], [14], [30]. Among these classifications, two dive shapes are commonly observed: square- and V-shaped dives, while the remaining dives are a variation of these core shapes. All of the classified dives in this study resembled one of the core dive shapes and differed either in skewness (ratio of ascent and descent rates), depth, duration, or the occurrence of wiggles. Previous studies have attempted to assign functions to different dive shapes by combining dive profiles, stomach-temperature telemetry, and swim speed to their analyses [14], [30]. These studies have suggested that skewed dives, with longer ascent or descent phases, may be attributed to a seal increasing the horizontal search component of the dive, or simply swimming along a bottom that progressively changes in depth (Lesage et al. 1999). Square-shaped dives have consistently been associated with foraging behavior as the predator spends more time at depth thus increasing the likelihood of encountering prey [14], [30], [43]. V-shaped dives may be related to exploratory behavior or travelling depending on the depth or skew of the dive respectively. Although multiple studies have attributed dive shapes to specific dive functions, some have suggested it is difficult to infer specific behaviors based on dive shape alone and that examining bouts of diving more accurately represents foraging behavior [1], [2]. Consequently, in this study dives were used to identify different bout types and were not compared individually.

### Diving Bouts

The behavior of harbor seals was organized into clusters or bouts of diving. In this study, only 12% of dives occurred outside of these bouts, likely the result of single dives related to haul-out behavior or underwater resting. Diving bouts differed primarily in depth and duration, but also in the percentage of square- and V-shaped dives occurring within the bout. These results are similar to those found in grey seals (*Halichoerus grypus*), Antarctic fur seals (*Arctocephalus gazella*), and harbor seals in other areas [1], [2], [16]. The differences in duration, dive shape, and percent of time spent at depth between bout types may suggest that each bout type represented a different behavior. Determining the exact nature of these behaviors remains difficult and limited [2], [36]; however, studies using stomach-temperature telemetry to identify feeding events, indicate that bouts of diving with a high percentage of time spent at depth (with a high percentage of square-shaped dives) are correlated with foraging activities [14], [36]. We identified one bout type with a high percentage of time spent at depth (Type I). The majority of dives occurring within this bout were square-shaped with 55% of the entire bout duration spent at depth (in the bottom portion of the dive). Wiggle dives have also been correlated with foraging behavior as vertical movement in the bottom portion of the dive can be attributed to movements within a prey patch [14]. Over 50% of the dives within both Type I and II bouts were classified as wiggle dives. This high percentage of wiggle dives suggests that both Type I and II bouts were used for foraging during this study.

Shallow diving bouts [2] as well as V-shaped dives [14] have been attributed to a number of different activities including traveling, predator avoidance, and exploration as animals are able to reduce drag and increase their chances of encountering prey by diving while traveling instead of swimming at the surface [14], [35], . In this study, Type II bouts contained mostly V-shaped dives; however, 44% of the dives were square-shaped. The mix of dive types may be associated with searching for and then feeding within prey patches located in shallow waters (<20 m) (Table 3). In contrast, Type I bouts had a higher proportion of time spent at depth (55%) and may be attributed to foraging in deeper waters (≥20 m). The majority of dives within Type I bouts were also square-shaped with wiggles, further indicating feeding within a prey patch. While both of these bout types potentially represent foraging behaviors, the differences between the two may provide insight into prey behavior. Type II bouts, with shallow, V-shaped dives and shorter dive durations, likely represented foraging within a tightly aggregated prey patch where the seal was able to find and capture prey near the surface with little time spent chasing through the water column. For Type I bouts, the differences in dive depth, duration, and bottom time suggest a different foraging strategy. Seals may have been foraging on more loosely aggregated or larger prey resulting in more time spent at depth in pursuit of prey.

### Behavioral Variation

Our model was built to analyze the influence of predictors on binomial, categorical data using a Bayesian framework. The structure can be applied to any type of categorical data and is applicable for many types of analyses. The Bayesian methodology used here makes it possible to exploit the basic elements of linear equations with Gaussian error as part of a more complex model [52]. The model was deliberately developed based on simplistic, categorical elements in order to demonstrate the applicability of this approach to behavioral studies. Time is one obvious predictor that was excluded from this analysis; future developments could translate this into a state-space model by including time as a continuous variable instead of using day/night as an indicator of time.

Model results indicate that dive behavior was significantly influenced by haul-out site, season, sex, and light; signifying that behavior was affected both spatially and temporally. Seals from Bird Rocks demonstrated a distinct change in the use of different bout types across seasons, while seals from Padilla Bay, located <20 km away, did not. In Washington State, the main return of pink salmon (*Oncorhynchus gorbuscha*) occurs in odd-numbered years, with only a very small return occurring in even years. Diet analysis in the Salish Sea indicates that harbor seals in 2005 (a pink salmon year) switched from consuming nearly 80% herring to 80% salmon at the end of July [20]. Multiple species of salmon return to the Salish Sea in July and August [53]. Certain species of salmon (pink, sockeye, and chum) typically remain at depths of 6–36 m as they return from the open ocean and make their way back to their natal streams [54]. These species also follow a diurnal vertical migration typically staying in the deeper end of their range during the day and moving into shallower waters at night. The first half of this study was conducted during a pink salmon year (2007), which leads us to conclude that the increase in shallow diving during the breeding season may have been correlated with an increase in salmon consumption during that time of year.

Sex affects diving behavior in harbor seals [6], [7] and due to the uneven sex ratio in this study the site differences we observed may also be correlated with sex. Female harbor seals forage throughout the breeding season, even while nursing [16], [30], and males tend to perform shallower dives while holding underwater breeding territories [7], [55]. More females were tagged in Padilla Bay than at Bird Rocks; however, this unbalanced sex ratio did not appear to be responsible for the increase in Type II bouts observed during the breeding season. At both sites, females performed a higher percentage of Type I bouts than any other bout type; however, the males at Padilla Bay performed significantly more Type II bouts during the breeding season (Figure 7). Notably, males from both sites performed a higher percentage of Type II bouts during the breeding season (females did not, Figure 7). Additionally, during the breeding season females performed a high percentage of Type I bouts than males, who seemed to favor Type II bouts. The increase in Type II bouts for males may be attributed to holding underwater breeding territories while they are attempting to mate. This pattern was visible at both haul-out sites, regardless of the number of females tagged, which suggests that sex was not the driving factor behind the behavioral differences observed between haul-out sites.

Harbor seals living within or near estuaries have a more diverse diet than those outside the estuary [20], [56]. In Padilla Bay, herring, salmon, and small schooling fish are consumed by harbor seals; however, seals also consume a number of benthic estuarine species such as gunnel (*Pholid spp.*), snake prickleback (*Lumpenus sagitta*), Pacific staghorn sculpin (*Leptocottus armatus*), plainfin midshipman (*Porichthys notatus*), and eelpout (*Zoarcid spp.*) [56]. Rocky-reef sites located just outside estuaries, likely used by estuarine seals, also show a more varied diet than that of rocky-reef sites away from estuaries [20]. The increased variety in diet near estuaries and the regular consumption of benthic estuarine prey within the estuary may be why Type I bouts are the predominant bout type used by Padilla Bay seals. Other studies of harbor seals from estuarine bays indicate that seals typically forage in the benthos and that no diurnal pattern in dive behavior is observed in those areas [12], [14], [16]. Individual seals may develop preferences for different foraging strategies, prey selection, or foraging locations [2] and seals in Padilla Bay likely exploit both the estuarine habitat and the seasonal increases in salmon abundance. Analysis of harbor seal movement patterns in the San Juan Islands shows that seals from Padilla Bay typically stayed within 5 km of the estuary during the breeding season, while seals from Bird Rocks made multiple trips >10 km from their tagging site [27]. Bird Rocks seals repeatedly traveled beyond the distance required to reach the estuary and traveled to rocky sites around the estuary, but were never documented to forage or haul-out within the estuary. Under this scenario, seals hauling-out in Padilla Bay foraged preferentially within the estuarine habitat. Foraging in the benthos throughout the year and exploiting the salmon run during the summer would explain why Type II bouts increased in abundance during the breeding season, but did not surpass the abundance of Type I bouts. Additionally, due to how the tags were programed (recording every 10 sec with the minimum dive depth of 5 m) we likely missed diving that occurred in shallow regions of the estuary. With different sampling protocols, we may have seen an even stronger signal of Padilla Bay seals preferentially foraging within the estuary, but we do not believe that our overall results would differ.

By analyzing foraging behavior using bouts instead of individual dives a more biologically relevant analysis was completed; however, details such as the difference between wiggle and non-wiggle square-shaped diving may have been excluded. Studies examining individual dives show that deep (≥20 m) square shaped dives without wiggles may indicate foraging in the benthos [14], [35], [44]. Benthic-dwelling prey are present in the harbor seal diet from rocky-reef sites, including Bird Rocks; however, a large majority of their diet is comprised of vertically-migrating schooling fish such as herring, Pacific hake (*Merluccius productus*), and salmon [20]. At Bird Rocks 26% of the dives during the non-breeding season were deep (≥20 m) and square-shaped without wiggles, potentially indicating benthic foraging. The use of both wiggle and non-wiggle square-shaped dives during the non-breeding season, before diurnally migrating salmon return to the Salish Sea, may be why such a high proportion of Type I bouts were used at this time of year and near equal proportions of Type I and II bouts during the breeding season.

### Conclusions

The variation in dive behavior over time that we observed suggests fluctuations in the predominant prey consumed by harbor seals. These variations may be related to annual migrations and movements of available prey in the study area. We were able to identify differences between two relatively close haul-out sites, which may allude to larger behavioral differences, such as prey specialization or habitat exploitation by different haul-out site groups. Prey specialization, with forage fish specialists and salmon specialists, has already been documented for harbor seals in southern Puget Sound [28] and likely explains the variation in diet and foraging behavior observed for harbor seals in this study [20], [50]. Bird Rocks and Padilla Bay showed similar variations in the use of different bout types; however, seals from Bird Rocks significantly changed the ratio of Type I to Type II bouts between seasons while seals from Padilla Bay did not. Bird Rocks seals dramatically increased the number of Type II bouts during the breeding season while Padilla Bay seals continued to use more Type I bouts. This change in behavior, which coincided with prey fluctuations in the region, suggests prey specialization between the two sites. Additionally, the increase in Type II bouts by males during the breeding season at both sites suggests that seals in both regions may maintain underwater breeding territories at or near foraging areas. These results provide a better understanding of the variability in harbor seal foraging behavior in the San Juan Islands and highlight the importance of examining behavioral variation on a small spatial scale. If all of the seals had been grouped together to examine San Juan Islands behavior as a whole, many of the influences we detected and the changes we observed may have been missed. This research sets up a baseline for understanding fine-scale behavioral variation and provides a reference point for documenting how seals may respond to ecosystem or prey fluctuations in the region.

Future studies should continue to document dive behavior at these sites to determine if these differences can be attributed to individual variability or to haul-out habitat exploitation. Additionally, if prey abundances in the region could be monitored concurrently with dive behavior, more concrete conclusions could be drawn regarding the link between prey fluctuations and dive behavior. If seals adjust their foraging strategies based on prey abundance, then their foraging behavior may be highly correlated with haul-out site habitat as seals will likely exploit prey located near their primary haul-out site. The data and analysis methods presented here provide an opportunity to continually monitor harbor seal diving to determine the relative importance of habitat characteristics on variations in foraging behavior. Comparing haul-out habitat to foraging area habitat may elude to either a correlation between foraging behavior and haul-out site type or to the degree of individual variability in foraging behaviors within the population. Either result will provide managers with important information regarding the foraging behavior of these predators and allow them to identify behavioral changes in the future.
